# Impact of low body mass index on reoperation risk and complications after joint arthroplasty: a cohort study

**DOI:** 10.1007/s00264-025-06518-z

**Published:** 2025-04-25

**Authors:** Pakpoom Ruangsomboon, Onlak Ruangsomboon, Ossama Al-Obaedi, Darius Luke Lameire, Daniel Pincus, Johnathan Robert Lex, Sebastian Tomescu, Bheeshma Ravi

**Affiliations:** 1 Sunnybrook Holland Orthopaedic and Arthritic Centre, Division of Orthopaedic Surgery, University of Toronto, Ontario, Toronto, Canada; 2 Faculty of Medicine, Siriraj Hospital, Mahidol University, Bangkok, Thailand; 3 Upstream Lab, MAP Centre for Urban Health Solutions, Li Ka Shing Knowledge Institute, Unity Health Toronto, Toronto, Ontario, Toronto, Canada; 4 Division of Orthopaedic Surgery, Department of Surgery, University of Toronto, Toronto, Canada; 5 Institute for Clinical Evaluative Sciences, Toronto, Canada

**Keywords:** Total hip arthroplasty, Total knee arthroplasty, BMI < 20, Low BMI, Low body mass index, Complication, Reoperation

## Abstract

**Purpose:**

The risks associated with low body mass index (BMI) in arthroplasty patients are underexplored. While outcomes of patients with elevated BMI are well-documented, low BMI patients may also face unique challenges, including malnutrition, osteopenia, and increased surgical risks and postoperative complications. To evaluate the impact of low BMI on reoperation risk and other complications compared with normal BMI among patients undergoing total hip or knee arthroplasty.

**Methods:**

This retrospective cohort study analyzed electronic health records of patients with BMI < 25 kg/m² who underwent hip or knee arthroplasty at Sunnybrook Holland Orthopaedic & Arthritic centre, Toronto, Canada between April 2, 2012, and April 6, 2023. Patients were stratified into low BMI (< 20 kg/m²) and normal BMI (20–24.9 kg/m²) groups, with their outcomes followed until November 2024. The main exposure was BMI categorized as low or normal. Other covariates controlled for were relevant demographics and comorbidities. The primary outcome was the risk of reoperation. The secondary outcome was composite complications (persistent pain, wound issues, and radiographic abnormalities). Survival analysis was performed with probabilities visualized with Kaplan-Meier curves. Multivariate Cox proportional hazards models were employed adjusting for potential confounders.

**Results:**

Among 1,162 included patients (mean [standard deviation] age, 68.8 [11.1] years; 70.1% women), 182 (15.7%) had low BMI and 980 (84.3%) had normal BMI. Kaplan-Meier curves demonstrated significantly higher risks of reoperation and composite complications in patients with low BMI compared to those with normal BMI (both *p* < 0.001). After adjusting for other covariates, low BMI was independently associated with increased risks of reoperation (adjusted Hazard Ratio (aHR), 5.8; 95% confidence interval (CI), 2.8–12.1; *p* < 0.001) and composite complications (aHR, 7.5; 95% CI, 3.9–14.5; *p* < 0.001).

**Conclusions:**

In this large cohort of arthroplasty patients, BMI < 20 kg/m² was associated with elevated risks of reoperation and composite complications. These findings emphasize the importance of tailored preoperative optimization and vigilant postoperative care for this high-risk population.

**Level of evidence:**

Level III.

**Supplementary Information:**

The online version contains supplementary material available at 10.1007/s00264-025-06518-z.

## Take home message

Patients with BMI < 20 kg/m² undergoing total hip or knee arthroplasty face significantly higher risks of reoperation and postoperative complications, such as persistent pain, wound issues, and radiographic abnormalities.

## Introduction

Body mass index (BMI) is a widely used measure to assess body composition and nutritional status that has a significant impact on health outcomes. While public health efforts have traditionally focused on the risks of obesity [1, 2], there is growing evidence that low BMI also poses serious health risks, particularly in patients with chronic illnesses [3–5]. Individuals with BMI < 20 kg/m², for instance, have been shown to experience increased mortality and exhibit higher rates of healthcare utilization, including consultations, prescriptions, and hospital admissions, compared to those with BMI of 20–24.9 kg/m² [6]. These findings highlight that even minor degrees of undernutrition may substantially increase morbidity and strain on healthcare resources.

In the context of orthopaedic surgery, patients with low BMI undergoing total joint arthroplasty (TJA) may face unique challenges. These include poor wound healing, lower bone quality, and an increased likelihood of complications, such as aseptic loosening or infections [7–10]. Regardless, available literature on TJA outcomes in low BMI patients is limited and presents mixed findings. While some studies suggest higher complication rates in underweight patients (BMI < 18.5 kg/m²), others report improvements in functional outcomes and quality of life, emphasizing the complexity of risk assessment in this population [11, 12]. Moreover, most previous studies focused on BMI cutoff for underweight at 18.5 kg/m², potentially overlooking the risks faced by patients with slightly higher, yet still low, BMI [7–16].

Given this gap of knowledge, redefining the low BMI threshold to < 20 kg/m² allows for a broader and more inclusive analysis of vulnerable patients. This expanded BMI cutoff can capture a larger proportion of patients who may be at risk but would otherwise not be included in studies using the traditional threshold of 18.5 kg/m² [7–16]. Also, this broader definition may better align with previous evidence suggesting that individuals with BMI < 20 kg/m² are already at elevated risk for adverse outcomes [14, 16], even in the absence of severe undernutrition [17, 18].

Therefore, this study aims to evaluate the impact of low BMI, defined as BMI < 20 kg/m², on the risks of reoperation and other complications following hip or knee arthroplasty. The study findings may be able to guide risk stratification and advise perioperative counseling and management in this potentially higher-risk patient population.

## Methods

### Study design and setting

This study was a single-center, retrospective cohort study conducted at Sunnybrook Holland Orthopaedic & Arthritic centre, the largest hip and knee arthroplasty center in Canada. The majority of the procedures performed at this facility are elective primary and revision hip and knee arthroplasty surgeries. Institutional electronic medical records from April 2, 2012, to April 6, 2023, were extracted for demographic, clinical, and procedural details, with patient outcomes followed until November, 30, 2024.

### Ethics approval

This study was approved by the local institutional review board (REB-CR6523).

### Funding declaration

This study was unfunded.

### Participants

The study included all patients with BMI < 25 kg/m² who underwent primary or revision hip or knee arthroplasty during the study period. Patients with missing outcomes or follow-up durations of less than 30 days were excluded. We stratified included patients into low BMI (BMI < 20 kg/m²) or normal BMI (BMI 20–24.9 kg/m²) group, with BMI calculated using height and weight recorded at the time of surgery.

### Data collection and outcomes

We retrieved patient demographics and comorbidities that effect arthroplasty outcomes, including diabetes mellitus, hypertension, alcohol use, smoking, osteoporosis, gastrointestinal disease, infectious disease, respiratory disease, cancer, genitourinary disease, neurological disease, thromboembolic disease, cardiac disease, haematologic disease, thyroid disorders, hepatobiliary disease, vascular disease, other endocrine disorders, and psychiatric conditions [19–21]. We also recorded procedural details, including the type of procedure (primary or revision) and joint involved (hip or knee). Reoperations and complications were also extracted from the institutional dataset.

The primary outcome was the risk of reoperation, defined as any subsequent surgical intervention following the index arthroplasty, including debridement of the surgical area, reduction of joint dislocation, periprosthetic fracture fixation, septic revision, aseptic revision and re-revision after revision arthroplasty. The secondary outcome was the risk of composite complications, defined as the occurrence of any one of the following: persistent pain longer than 6 months [22–24], wound complications [25], and radiographic changes indicative of implant failure, loosening, periprosthetic fracture or dislocation [26–28]. Wound complication, as delineated by the Surgical Infection Study Group (SISG), includes the manifestation of pain, edema, erythema, warmth, and compromised function surrounding the surgical site [25].

### Statistical analysis

Categorical variables were summarized as frequencies and percentages and compared using the Chi-squared or Fisher’s Exact test, as appropriate. Continuous variables with normal distribution were presented as mean with standard deviation and compared using the Student’s t-test, while those with non-normal distribution were reported as median with interquartile range (IQR).

For the primary analysis, time-to-event outcomes were visualized using Kaplan-Meier survival curves, and statistical significance was assessed using the log-rank test, comparing the outcomes between the low and normal BMI categories. Furthermore, multivariate Cox proportional hazards regression models were constructed to evaluate the association between BMI categories and study outcomes. Covariates for the multivariate model were initially selected based on expert opinion and literatures [19, 29]. They included sex, age at surgery, and all comorbidities that could affect arthroplasty outcomes previously specified. We then selected the covariates to be included in the final model using univariate Cox regression analyses, with variables showing *p*-values < 0.2 included in the final model, to avoid model overfitting arising from the low number of outcome events. The proportional hazards assumption for the final model was assessed using Schoenfeld residuals [30]. Results of the multivariate models are presented as adjusted hazard ratios (aHRs) with 95% confidence intervals (CIs), with a two-sided *p*-value < 0.05 considered statistically significant.

Kaplan-Meier survival analyses were also performed in a-priori subgroups, with the cohort stratified by procedure type (primary or revision) and joint type (hip or knee). Moreover, to minimize confounding and confirm the robustness of the findings, propensity score matching in the study population for Kaplan-Meier survival analyses was performed as a sensitivity analysis. Propensity scores were calculated based on baseline characteristics identified from existing literature as previously elaborated. A nearest neighbor matching method with a caliper of 0.2 based on the logit of propensity scores was employed to balance baseline characteristics between BMI cohorts. Matching was conducted in both the entire study cohort and separately for each procedure type in the subgroup analysis where applicable to balance baseline characteristics. The balance between matched cohorts was assessed by examining absolute standardized differences (ASD), with values < 0.2 indicating adequate balance [31]. We did not perform the Cox proportional hazards regression analyses in the subgroups due to rare outcome events in the subgroups posing a very high risk of model overfitting.

Statistical analyses were performed using R software (version 4.4.2), with the MatchIt, Survival, and Survminer packages utilized for propensity score matching, survival analyses, and Kaplan-Meier visualization [32–34].

## Results

### Demographic and baseline characteristics

A total of 1,162 patients were included in the analysis. Their mean age was 68.8 ± 11.1 years, and 70.1% were women. Of these, 182 patients (15.7%) had a low BMI, while 980 patients (84.3%) had a normal BMI. Baseline characteristics by BMI category are summarized in Table 1. The low BMI group had a higher proportion of men patients (77.5% vs. 68.7%, *p* = 0.022), a lower proportion of genitourinary disease (4.9% vs. 10.0%, *p* = 0.043), and a higher prevalence of respiratory disease (21.4% vs. 14.8%, *p* = 0.032) and smoking (6.0% vs. 2.4%, *p* = 0.018) compared to the normal BMI group. The median follow-up time of the study population was 2.6 yeasts (IQR 1.4–4.3 years), with a maximum follow-up time of 12.4 years.

**Table 1:** Baseline demographic and clinical characteristics stratified by BMI category

	Low BMI (*n* = 182)	Normal BMI (*n* = 980)	*p*-value
Men	141 (77.5%)	673 (68.7%)	0.022
Age at surgery (years)	67.7 *±* 12.2	69.0 *±* 10.9	0.177
Alcohol consumption	2 (1.1%)	22 (2.2%)	0.475
Gastrointestinal disease	43 (23.6%)	283 (28.9%)	0.174
Infectious disease	2 (1.1%)	7 (0.7%)	0.934
Respiratory disease	39 (21.4%)	145 (14.8%)	0.032
Cancer	3 (1.6%)	23 (2.3%)	0.755
Genitourinary disease	9 (4.9%)	98 (10.0%)	0.043
Neurological disease	18 (9.9%)	75 (7.7%)	0.383
Thromboembolic disease	0 (0%)	3 (0.3%)	1
Cardiac disease	31 (17%)	229 (23.4%)	0.074
Hematologic disease	13 (7.1%)	55 (5.6%)	0.525
Thyroid disease	19 (10.4%)	108 (11.0%)	0.919
Hepatobiliary disease	3 (1.6%)	19 (1.9%)	1
Vascular disease	3 (1.6%)	19 (1.9%)	1
Smoking	11 (6.0%)	24 (2.4%)	0.018
Osteoporosis	12 (6.6%)	51 (5.2%)	0.561
Other Endocrine disease	28 (15.4%)	123 (12.6%)	0.356
Psychiatric disease	16 (8.6%)	57 (5.8%)	0.176
Diabetes mellitus	12 (6.6%)	87 (8.9%)	0.385
Hypertension	32 (17.6%)	127 (13.0%)	0.121

Note: data presented as frequency (%) or mean *±* standard deviation

Abbreviation: BMI, body mass index

### Effect of BMI on the risks of reoperation (primary outcome) and composite complications (secondary outcomes)

Patients with low BMI experienced higher rates of adverse events compared to those with normal BMI (Table 2). Reoperation occurred in 7.7% of low BMI patients versus 1.6% in the normal BMI group. Composite complications were also more frequently seen in the low BMI cohort (11.5% vs. 1.6%). Details of the composite components found among the two groups are described in Table 2.

**Table 2:** Rate of primary and secondary outcomes stratified by BMI category

Outcome	Low BMI (*n* = 182)	Normal BMI (*n* = 980)
Reoperation	14 (7.7%)	16 (1.6%)
Composite complications	21 (11.5%)	16 (1.6%)
- Persistent pain	17 (9.3%)	2 (0.2%)
- Wound complications	4 (2.2%)	1 (0.1%)
- Radiographic changes	14 (7.7%)	16 (1.6%)

Note: data presented as frequency (%)

Abbreviation: BMI, body mass index

**Fig. 1:**
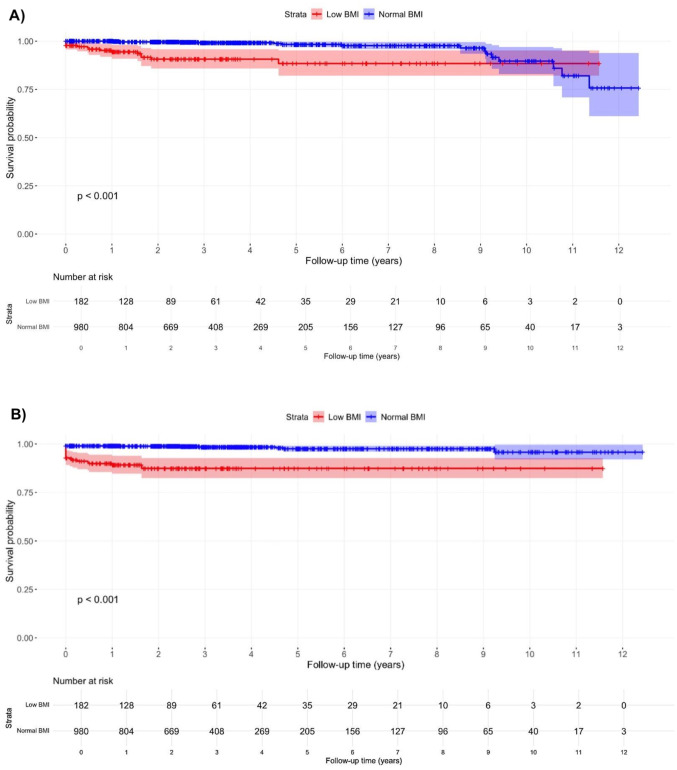
Kaplan-Meier survival curves for risk of reoperation (**A**) and composite complications (**B**) after total joint arthroplasty by BMI category. Note: Low BMI: BMI < 20 kg/m^2^, Normal BMI: BMI 20–24.9 kg/m^2^. Abbreviation: BMI, body mass index

The Kaplan-Meier survival curves demonstrate that patients with low BMI had a significantly higher risk of reoperation compared to those with normal BMI (Fig. 1A, *p* < 0.001). Multivariate Cox regression analysis showed that low BMI was associated with a 5.8-fold higher risk of reoperation (aHR: 5.8, 95% CI: 2.8–12.1, *p* < 0.001). The other independent predictor for reoperation was age at surgery (HR: 0.96, 95% CI: 0.93–0.99, *p* = 0.006).

For composite complications, the Kaplan-Meier curves also indicate that patients with low BMI had a significantly higher risk of composite complications than those with normal BMI (Fig. 1B, *p* < 0.001). Concordantly, low BMI was the only significant predictor in the final multivariate Cox regression model, associated with a 7.5-fold increased risk of composite complications (aHR: 7.5, 95% CI: 3.9–14.5, *p* < 0.001).

Details of propensity score matching, including baseline characteristics and ASD metrics, are provided in Supplementary Table 1. We did not find any imbalances in characteristics between the low and normal BMI groups within the whole study population that need to be matched; therefore, no further sensitivity analyses were performed for the whole population.

### Survival analyses by procedure type

**Fig. 2:**
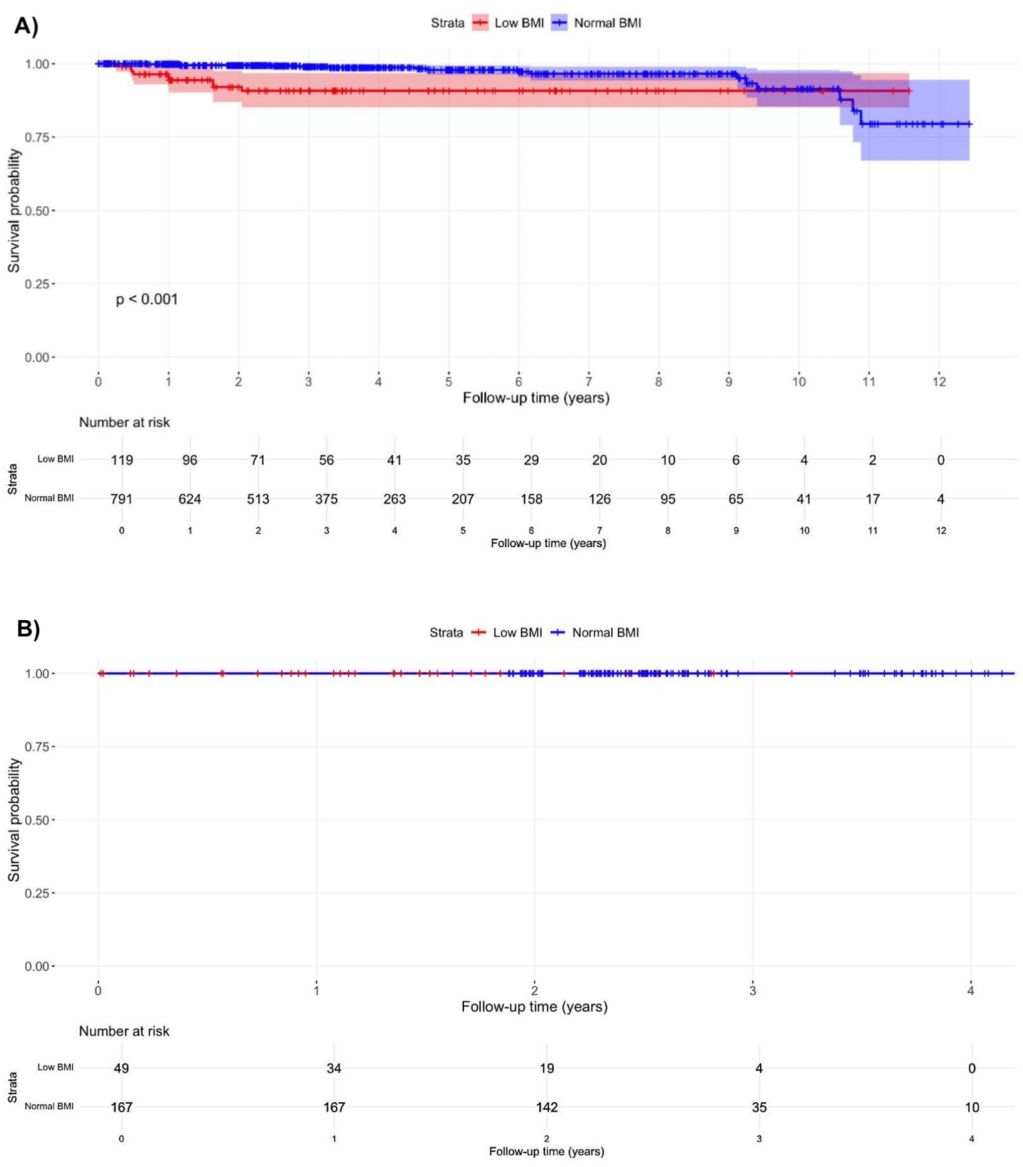
Procedure-specific Kaplan-Meier survival curves for risk of reoperation after total knee arthroplasty (**A**) and total hip arthroplasty (**B**) by BMI category. Note: Low BMI: BMI < 20 kg/m^2^, Normal BMI: BMI 20–24.9 kg/m^2^. Abbreviation: BMI, body mass index

Kaplan-Meier curves stratified by procedure type for reoperation risk are depicted in Figs. 2. For primary knee arthroplasty, patients with low BMI had a significantly higher risk of reoperation compared to those with normal BMI (Fig. 2A, *p* < 0.001). In primary hip arthroplasty, no reoperation events were observed, precluding statistical comparisons (Fig. 2B). For revision hip and knee arthroplasty, the sample size for each subgroup was less than 20 at baseline, so we did not perform the subgroup analysis due to inadequate statistical power.

**Fig. 3:**
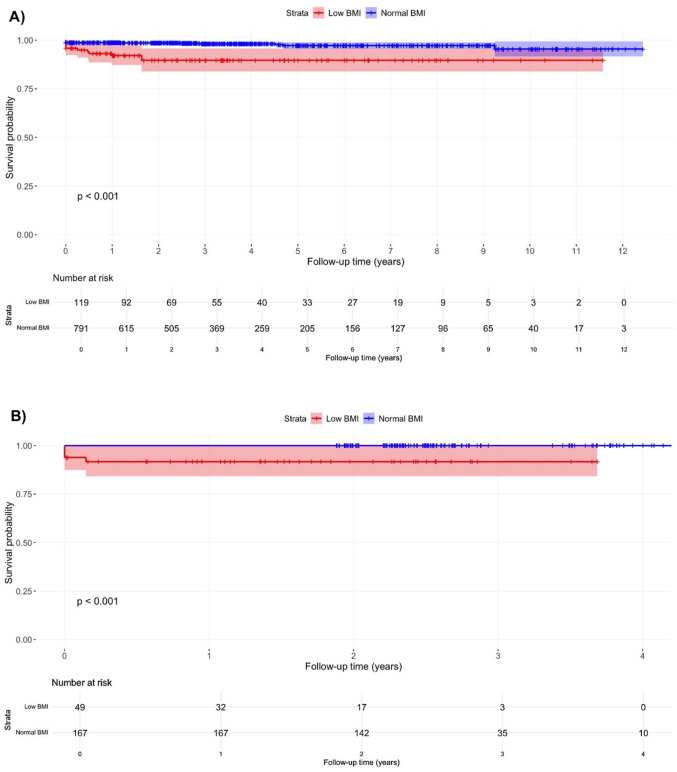
Procedure-specific Kaplan-Meier survival curves for composite complications after total knee arthroplasty (**A**) and total hip arthroplasty (**B**) by BMI category. Note: Low BMI: BMI < 20 kg/m^2^, Normal BMI: BMI 20–24.9 kg/m^2^. Abbreviation: BMI, body mass index

Kaplan-Meier curves for composite complications stratified by procedure type provide further insights into procedure-specific risks (Figs. 3). Patients with low BMI exhibited a significantly higher risk of composite complications for both primary knee arthroplasty (Fig. 3A, *p* < 0.001) and primary hip arthroplasty (Fig. 3B, *p* < 0.001). Similar to the analysis for the primary outcome, there were inadequate number of participants undergoing revision hip and knee revision arthroplasty to allow for appropriate and meaningful log-rank comparisons; therefore, they were not performed.

After matching for baseline characteristics specific to each procedure type, the associations between low BMI and risks of reoperation and composite complications remained consistent with the primary analyses for both primary knee and primary hip arthroplasty (Supplementary Fig. 1A-1B and 2 A-2B).

## Discussion

This study demonstrates that patients with BMI < 20 kg/m² undergoing TJA face significantly higher risks of reoperation and composite complications compared to those with normal BMI. These associations persisted across most primary procedures, with primary knee arthroplasty showing the greatest risk elevation. Interestingly, while prior guidelines often classify BMI < 18.5 kg/m² as underweight [35], our findings highlight that even patients with BMI between 18.5 and 20 kg/m² —a range still considered “normal”—are also vulnerable to adverse outcomes [36]. This finding supports the expanded definition of at-risk BMI thresholds associated with TJA outcomes. It also emphasizes the importance of preoperative counseling to set realistic expectations and the potential role of pre-operative nutrition programs for these patients to optimize their outcomes.

Our findings align with existing literature that highlighted elevated complication risks in patients with low BMI using the 18.5 kg/m² threshold. Alfonso et al. identified elevated risks of dislocation, infection, and prolonged hospital stays in underweight patients undergoing total hip arthroplasty (THA) [8]. Zalikha et al. also reported higher complication rates and longer hospital stays in underweight patients undergoing revision arthroplasty [37]. As previously discussed, these studies often relied on the “underweight” classification as BMI < 18.5 kg/m², potentially overlooking patients with BMI in the 18.5–20 kg/m² range [7, 8, 12, 16, 38]. By redefining the low BMI threshold to < 20 kg/m², our study expands upon this prior evidence, offering a more inclusive perspective on risk stratification.

Previous studies evaluating low BMI patients with the 20 kg/m² threshold further reinforce our findings. McDonald et al. reported increased odds of revision, sepsis, and periprosthetic fractures in patients with BMI < 20 kg/m² undergoing THA [16]. Similarly, Katakam et al. observed that patients with BMI < 20 kg/m² had an eight fold increase in mortality risk following TJA [14]. However, many of these studies and other prior research hardly focused on hip procedures or specific postoperative timeframes [39, 40]. While some studies, such as Shaparin et al., highlighted early postoperative complications in underweight patients, our findings emphasize sustained risks towards mid-term follow-up period, thereby addressing a key gap in the literature [39]. By incorporating a broader cohort, longer follow-up time, and procedure-specific analyses, our study provides a more nuanced understanding of the relationship between low BMI and arthroplasty outcomes. Nevertheless, it is important to note that we did not specifically assess and compare low BMI ranges due to the small sample size in these ranges, and perhaps the revisions encountered were primarily due to patients with a BMI < 18.5 kg/m² rather than among those with a BMI between 18.5 and 20 kg/m². This leaves a gap of knowledge for future larger studies to explore the risks of adverse outcomes across these low BMI ranges.

While the elevated risks associated with low BMI are consistent with prior research, there remains variability in outcomes across studies, which could have stemmed from population-specific and methodological differences. For example, while Kwon et al. observed worse functional outcomes in underweight total knee arthroplasty patients, they found no significant difference in complication rates [7]. The discordance in complication rates between their study and ours was likely due to different patient population and outcome definition. Kwon et al. included a relatively older patient group, employed the 18.5 kg/m² cutoff, and did not include pain as an independent outcome measure. Interestingly, we found low BMI patients to have encountered a largely higher incidence of persistent pain among other components of the composite complication compared to the normal BMI group. This finding could have been because low BMI patients tend to have thinner subcutaneous fatty soft tissue [41], thus increasing the likelihood of hardware irritation causing them to experience pain.

In addition, the observed differences in outcomes between the two BMI categories by procedure type in our study may reflect the interplay of anatomical, biomechanical, and institutional factors. In primary knee arthroplasty, low BMI patients faced increased risks of reoperation and composite complications, likely due to thinner soft tissue coverage, and reduced natural barriers to infection, all of which could exacerbate wound healing and increase infection risks [15, 42, 43]. Notably, the Kaplan-Meier curves suggest that once the recovery plateau has passed at around ten years post-surgery, low BMI patients still maintained a relatively constant mechanical failure rate compared to normal BMI patients, probably due to lower mechanical joint loads in the low BMI group. Conversely, the lack of significant associations in primary hip arthroplasty may reflect a recovery advantage intrinsic to the hip joint and the mitigating effects of the high surgical volume at our centre [44–46]. For revision procedures, the number of patients and outcome events were too low to merit meaningful statistical analyses. This underscores the need for larger, population-based studies to validate the impact of BMI < 20 kg/m² in revision arthroplasty and its interplay with confounding clinical variables.

### Strengths and limitations

This study’s key strengths include its large, diverse cohort, prolonged mid-term follow-up, and comprehensive analysis across multiple procedure types. These factors enhance the generalizability of our findings to different real-world clinical settings. Additionally, the use of propensity score matching in our sensitivity analyses helps ensure the robustness of our results. However, several limitations warrant consideration. First, the study was not powered for longer-term outcomes, limiting our ability to assess risks beyond the mid-term follow-up period. Second, potential residual confounding, such as unmeasured nutritional status [47, 48], muscle mass [49], and ethnicity [50, 51], might have influenced the results. Third, some of our subgroup and sensitivity analyses could not be performed as intended due to inadequate statistical power, such as the procedure-specific analyses for hip and knee revision surgeries. Finally, the lack of granular data on perioperative decision-making, such as surgeon-specific experience and intraoperative factors (e.g., surgical approach and prosthesis selection) or environmental factors (e.g., logistic system of operating theatre, laminar airflow [52, 53], and sequence of operating schedule [54–56]) might have contributed to the variability in procedure-specific outcomes. For instance, the lack of reoperation risk differences in primary hip arthroplasty might have partially resulted from surgical expertise or perioperative management decisions that were not captured in this analysis.

## Conclusion

This study highlights the elevated risks of reoperation and complications in patients with BMI < 20 kg/m² undergoing TJA. Our results emphasize the need to re-evaluate BMI-based risk stratification thresholds. By broadening the focus beyond the conventional “underweight” classification of BMI < 18.5 kg/m², our findings underscore the importance of tailored preoperative counseling and perioperative management to improve outcomes for this vulnerable population. These insights are critical for informing clinical decision-making and optimizing care strategies in arthroplasty patients.

## Electronic supplementary material

Below is the link to the electronic supplementary material.

Supplementary Material 1

Supplementary Material 2

Supplementary Material 3

## Data Availability

Requests for data not shown in the body of this manuscript can be made to the corresponding author.
