# Supplementary material for: Impact of low body mass index on reoperation risk and complications after joint arthroplasty: a cohort study
Source: Int Orthop. 2025 Apr 25;49(7):1587–97. doi: 10.1007/s00264-025-06518-z (PMC12179008; doi:10.1007/s00264-025-06518-z)
Supplement: Supplementary file 1 — Supplementary Material 1 [file 264_2025_6518_MOESM1_ESM.docx]

**Supplementary Table 1. Characteristics evaluated for propensity matching between the low and normal BMI groups**


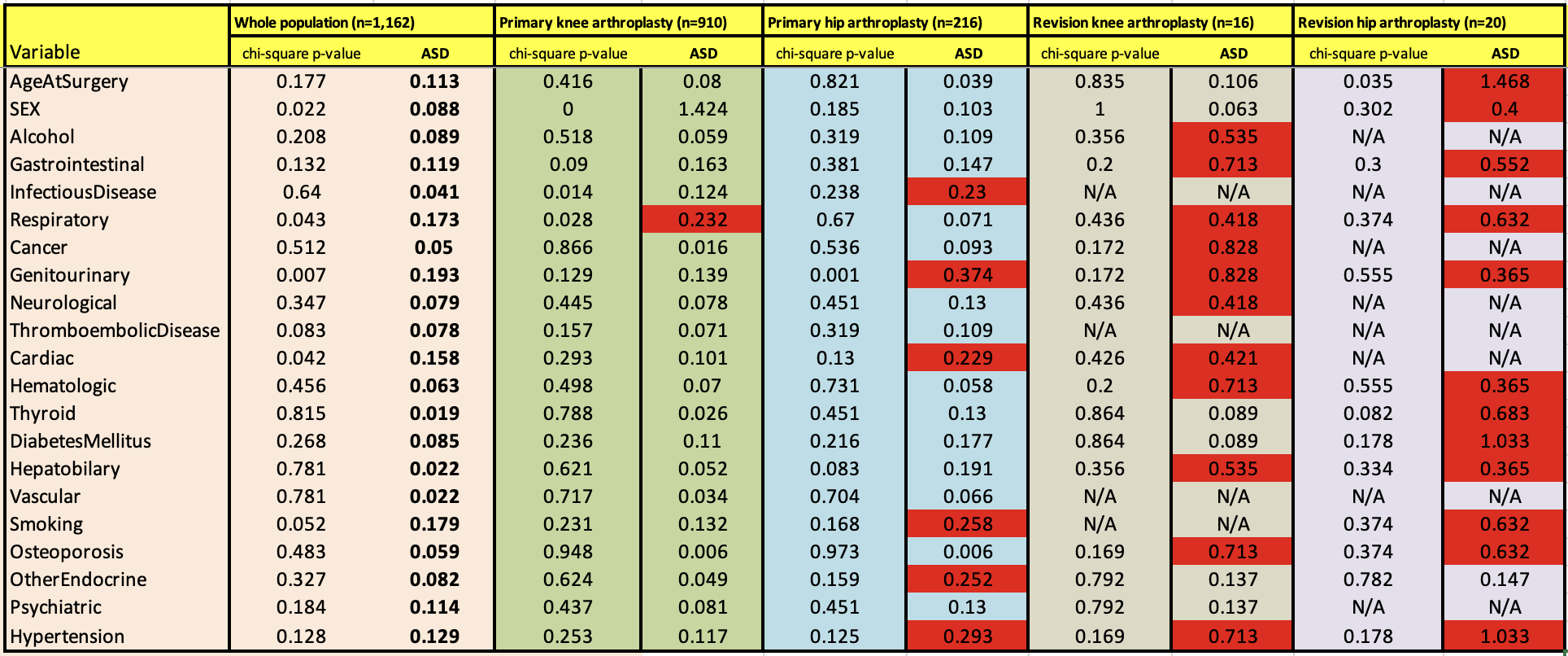


Note: characteristics highlighted in red were matched.

Abbreviation: ASD, absolute standardized differences.
