# Supplementary material for: Impact of low body mass index on reoperation risk and complications after joint arthroplasty: a cohort study
Source: Int Orthop. 2025 Apr 25;49(7):1587–97. doi: 10.1007/s00264-025-06518-z (PMC12179008; doi:10.1007/s00264-025-06518-z)
Supplement: Supplementary file 3 — Supplementary Material 3 [file 264_2025_6518_MOESM3_ESM.docx]

**Supplementary Figure 2. Procedure-specific Kaplan-Meier survival curves for composite complications after total knee arthroplasty (A) and total hip arthroplasty (B) by BMI category after propensity matching**

**A.**
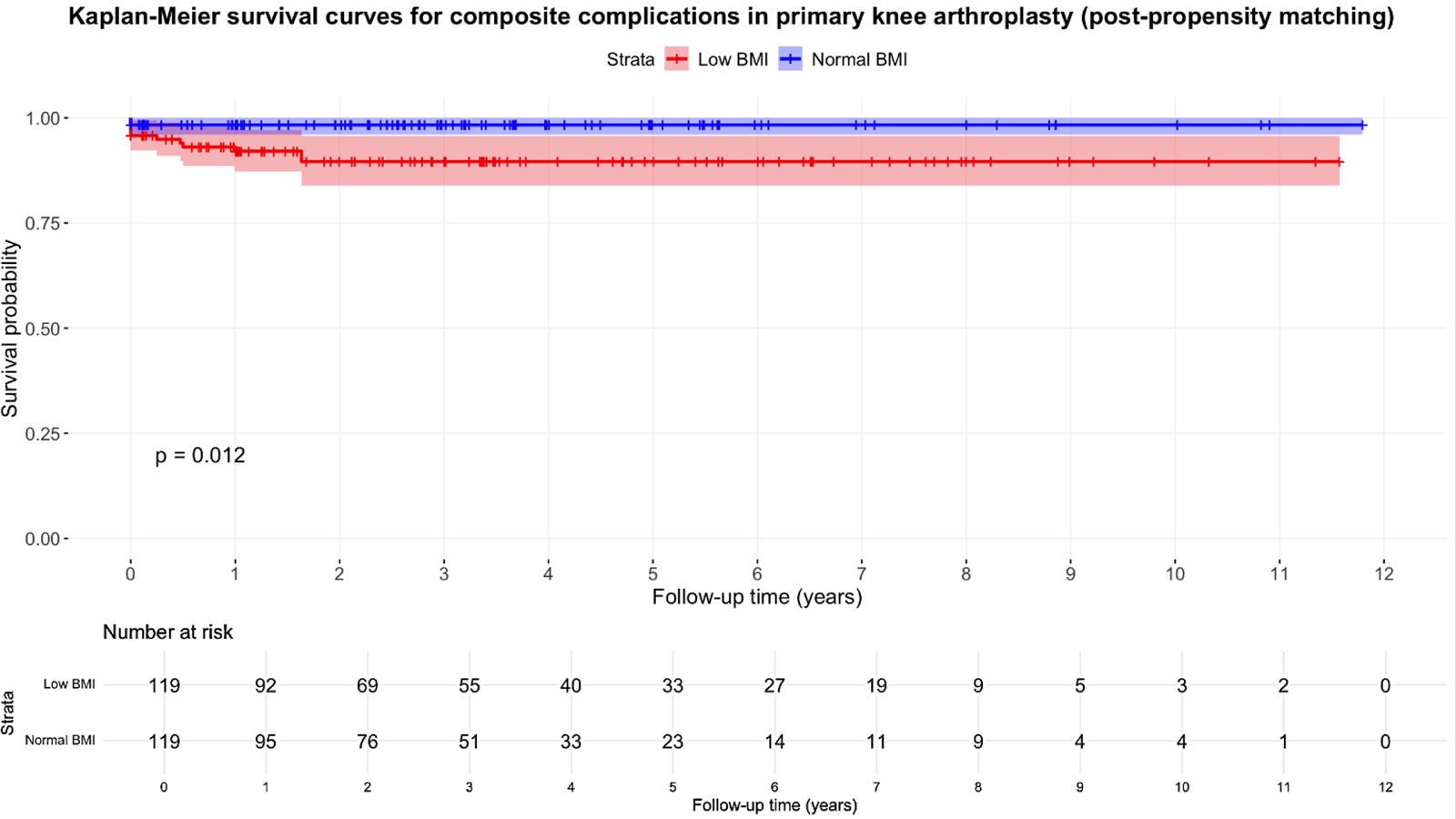
**B.**
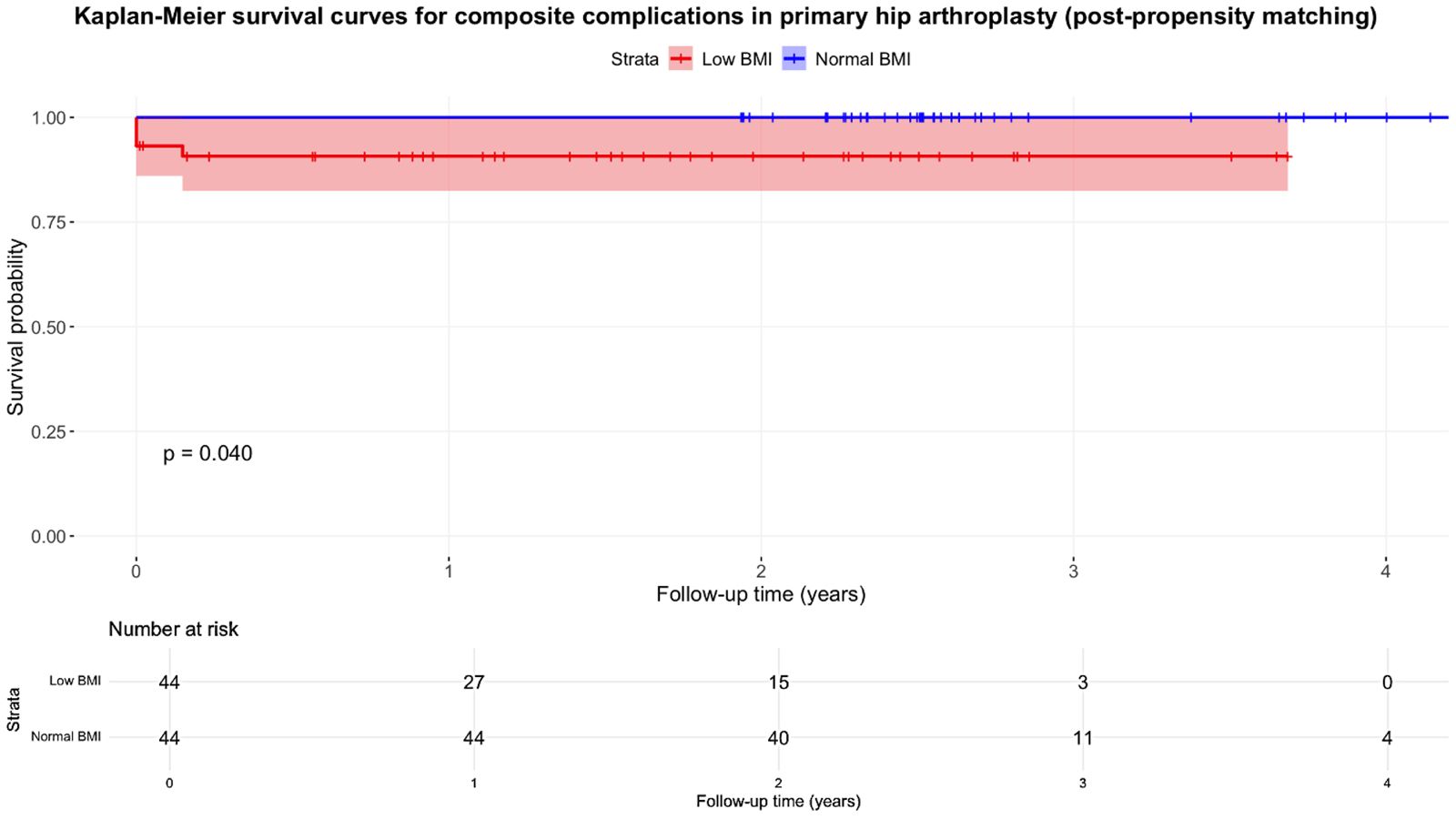


Note: Low BMI: BMI <20 kg/m^2^, Normal BMI: BMI 20-24.9 kg/m^2^

Abbreviation: BMI, body mass index.
